# Uncertainty in Environmental Micropollutant Modeling

**DOI:** 10.1007/s00267-024-01989-z

**Published:** 2024-05-30

**Authors:** Heidi Ahkola, Niina Kotamäki, Eero Siivola, Jussi Tiira, Stefano Imoscopi, Matteo Riva, Ulas Tezel, Janne Juntunen

**Affiliations:** 1 Finnish Environment Institute (Syke), Latokartanonkaari 11, 00790 Helsinki, Finland; 2 IDSIA, Università della Svizzera italiana (USI), Via Buffi 13, 6900 Lugano, Switzerland; 3 Independent Researcher. Work Carried Out While Employed at IDSIA, USI, Lugano, Switzerland; 4 Institute of Environmental Sciences, Boğaziçi University, Hisar Campus, Bebek, Istanbul, 34342 Turkey

## Abstract

Water pollution policies have been enacted across the globe to minimize the environmental risks posed by micropollutants (MPs). For regulative institutions to be able to ensure the realization of environmental objectives, they need information on the environmental fate of MPs. Furthermore, there is an urgent need to further improve environmental decision-making, which heavily relies on scientific data. Use of mathematical and computational modeling in environmental permit processes for water construction activities has increased. Uncertainty of input data considers several steps from sampling and analysis to physico-chemical characteristics of MP. Machine learning (ML) methods are an emerging technique in this field. ML techniques might become more crucial for MP modeling as the amount of data is constantly increasing and the emerging new ML approaches and applications are developed. It seems that both modeling strategies, traditional and ML, use quite similar methods to obtain uncertainties. Process based models cannot consider all known and relevant processes, making the comprehensive estimation of uncertainty challenging. Problems in a comprehensive uncertainty analysis within ML approach are even greater. For both approaches generic and common method seems to be more useful in a practice than those emerging from ab initio. The implementation of the modeling results, including uncertainty and the precautionary principle, should be researched more deeply to achieve a reliable estimation of the effect of an action on the chemical and ecological status of an environment without underestimating or overestimating the risk. The prevailing uncertainties need to be identified and acknowledged and if possible, reduced. This paper provides an overview of different aspects that concern the topic of uncertainty in MP modeling.

## Introduction

The scientific community has expressed concern over the chemicalization of the environment and its effects on human health (Sobek et al. 2016, Sjöström and Talanquer 2018). Chemicalization has been recognized to be one of the drivers causing biodiversity loss (Groh et al. 2022). Anthropogenic actions impact on environmental chemical concentrations, for example, via the discharging the wastewater into rivers (i.e., effluent) or through pollutants released into the atmosphere via air emissions. Micropollutants (MPs) comprise a broad group of anthropogenic and natural chemicals, such as those used in personal care products, pharmaceuticals, pesticides, and industrial chemicals. Due to the widespread utilization of MPs, they end up in various water bodies from different sources (Tong et al. 2022). Generally, MPs can bioaccumulate and be toxic to living organisms. As there are always many MPs present in the environment the discussion is shifting from individual chemicals to combined or mixture effects (Beyer et al. 2014, Panizzi et al. 2017, Shao et al. 2019, Müller et al. 2020).

Water pollution policies have been enacted across the globe to minimize the environmental risks posed by MPs (Rockström et al. 2009). The Water Framework Directive (WFD; EC 2000), legislated in the European Union (EU), requires all member states to achieve and maintain a good chemical and ecological status for all water bodies within their territories. This has led the EU member countries to implement ambitious monitoring, planning, and water management frameworks to achieve the *good status* objective. Recently, also the private sector has faced increased pressure to consider the WFD objectives. This was highlighted in the 2015 Weser ruling (Weser ruling 2015) by the Court of Justice of the European Union (CJEU). The court stated that the environmental objectives outlined in the WFD are also legally binding on industrial facilities. Consequently, any new organization applying for an environmental permit must demonstrate that its operations will not negatively impact any water quality indicator. These same requirements apply to projects related to remediation of watercourses. Producing data on the impact and environmental fate of MPs is crucial. This is important for regulative institutions to ensure that the environmental objectives are carefully considered. However, measuring organic MP concentrations with instant grab water samples is expensive due to laborious chemical analysis which can take several weeks. To expand the dataset by taking grab water samples more frequently would further increase the chemical analysis costs. This situation underscores the need for reliable and cost-effective environmental assessment and has led to the increased utilization of mathematical and computational modeling in environmental management processes and especially in permit processes.

In a management of chemical emerged issues there is a need for a model-based approach at different spatial and temporal scales, from global (the entire world) to local (a single river or sub-catchment area), and from timescales of less than a day to decadal timescales (Kroeze et al. 2016). Furthermore, when predicting the environmental behavior of new substances for which there is no environmental concentration data or release information, modeling can be employed in the regulatory risk assessment (Van de Meent et al. 2011). Emerging safe and sustainable by design framework is using environmental fate modeling to design new, less hazardous chemical compounds than the previous ones (van Dijk et al. 2022).

On a conceptual level, modeling the environmental fate of chemicals is straightforward, as all the processes that determine the environmental fate of chemicals are—in principle—well-known (Tong 2022). However, conventional modeling approaches (i.e., deterministic modeling and parametric modeling) are particularly challenging when attempting to factor in all known processes affecting chemicals. In principle rejecting any of them causes structural uncertainty. Accounting for all these processes would require knowledge of several elements, including fish population, vegetation, the distribution of certain microbes, and other processes or parameters that affect chemical transformation processes. Another challenge is the mismatch between the timescales associated with conventional modeling methods and knowledge on the distribution of chemicals. The partition coefficients of a chemical between major phases (e.g., water, air, and land) are expressed in equilibrium conditions, but hydrodynamical models solve equations in time scales of seconds. In summary, the combined uncertainties involved in traditional modeling the impact and environmental fate of MPs are massive.

**Fig. 1:**
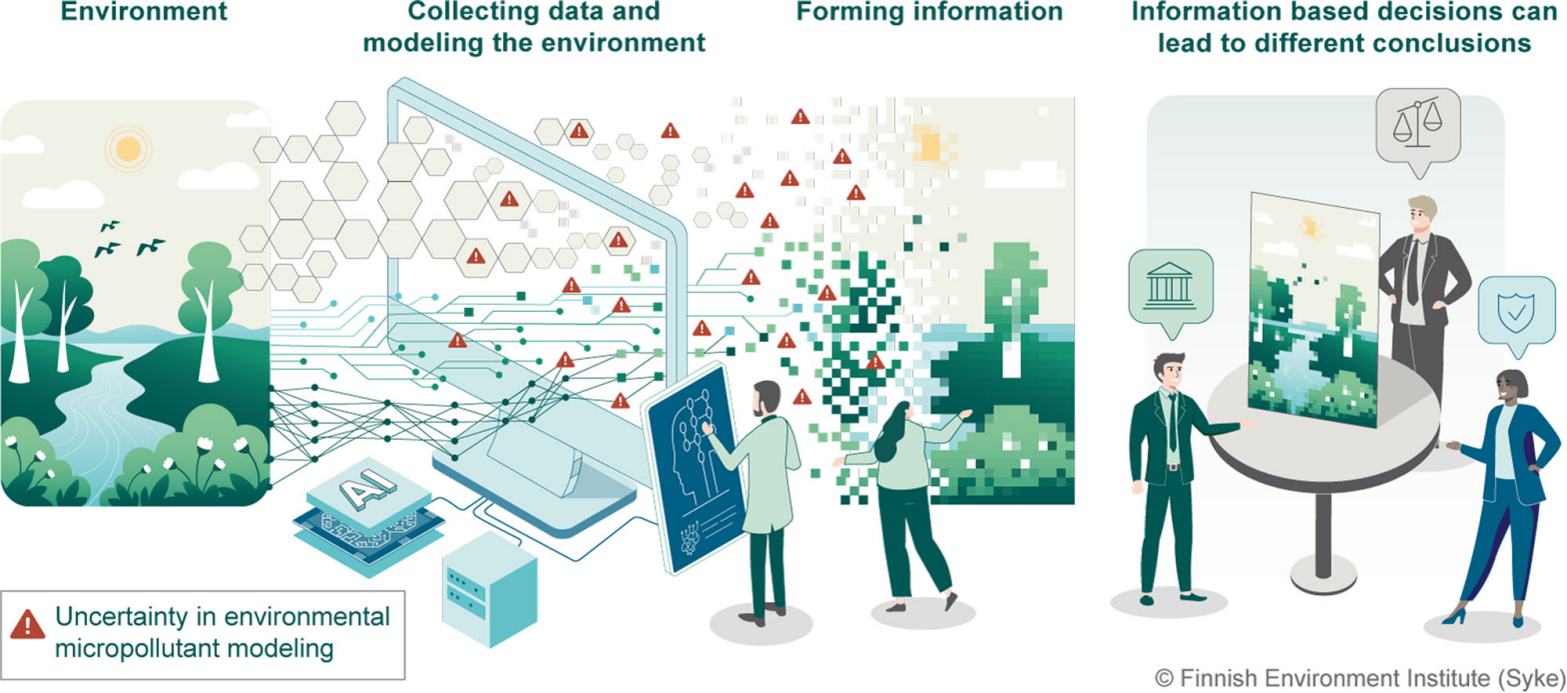
Conceptual flow of uncertainty in decision making

Modeling can be considered as an effective tool in assessing effects of e.g., water construction activities. To meet the increasing demand for modeling, the effects of these uncertainties on modeling results must be considered (Fig. 1). Uncertainty of modeling is partially dependent of the quality of the initial, input and training data used in the models (Kläs and Vollmer 2018). This study discusses the uncertainties in MP modeling and addresses the science–policy connection with respect to MPs and the role of emerging machine learning (ML) approaches in improving the reliability of modeling. These issues are critical when considering the use of MP modeling in environmental decision-making. In this paper we aim to recognize and discuss about uncertainties which authors find relevant.

## The policy context: Importance of data and models

### EU Micropollutant Regulations

Robust water management policies, processes, and guidelines, established through national or multinational legislation, form the basis for the protection, management, and sustainable use of water resources. In this study, we focus on the European Union (EU) where the Water Framework Directive (WFD, EC 2000) primarily enacts environmental water policy objectives. These are further supported by the Environmental Quality Standards Directive (EC 2008) and the Groundwater Directive (EC 2006a). The WFD mandates all EU member states to achieve and maintain good chemical (and ecological) status for their surface waters and groundwaters. The identification of priority chemicals is governed by the Priority Substances Directive (EC 2008, EU 2013), which is a component of the WFD. At present, the EU bases its chemical status classification on 45 priority pollutants. This list of priority chemicals is reviewed every 6 years against potentially concerning substances presented in the Watch List, established in 2015 and updated in 2018 and 2020. EU member states must monitor Watch List substances at least once a year for 4 years to assess the potential risks posed by these substances. Other more detailed directives, such as the Sustainable Use of Pesticides Directive (EC 2009a), require more specific knowledge. Each member state must also identify whether a substance is of national or local concern. At the EU level, the use of harmful substances is prohibited or restricted by several regulatory ordinances: the Registration, Evaluation, Authorization and Restriction of Chemicals (REACH) Regulation (EC 2006b), which applies directly to industry and manufacturing activities; the Persistent Organic Pollutants (POP) Regulation (EC 2019), which bans or restricts the use of POPs in chemical products and articles; and legislation on plant protection products (PPPs) (EC 2009b).

EU water policies are implemented at two primary levels. At the national level, member states are obligated to monitor and assess the status of their waters. If necessary, they are required to take action to improve the status. At the local level, the EU water policies are implemented through mechanisms such as the Environmental Impact Assessment (EIA) Directive 2014/52/EU (EC 2014). This directive provides a framework for assessing the potential environmental impacts of proposed projects. The EIA evaluation is typically conducted prior to the initiation of the actual environmental permit process. This ensures that potential environmental impacts, including those related to chemical pollutants, are thoroughly assessed before any project or development proceeds.

### Modeling and Monitoring Requirements

Environmental decision-making relies heavily on scientific information. Mathematical modeling is considered as a useful tool for providing understanding on the transport and environmental fate of MPs and the impact of these chemicals on aquatic ecosystems and, ultimately, on the welfare of humans. However, reliable modeling requires an abundance of data, and there are significant limitations to the monitoring of MPs and their effects. The monitoring data requirements necessary for national-scale assessments, where a coarse temporal frequency may be adequate, differ from those required for individual permit processes. For these processes, local high-resolution spatiotemporal information is essential for a reliable impact assessment. The data generated by monitoring programs for national-level chemical status assessments may not be sufficient for conducting local impact assessments. This highlights the need for more detailed, localized data collection in order to accurately assess the environmental impact at a local level. Furthermore, the physical characteristics of waterways and water chemistry characteristics such as pH, electrical conductivity, turbidity, total organic carbon (TOC), dissolved organic carbon (DOC) or nutrients are studied more extensively than concentrations of organic MPs. For example, water flow and water chemistry characteristics can be monitored sub-daily resolution using web-enabled monitoring sensors. Furthermore, pollution monitoring and management efforts have focused on conventional priority substances (e.g., PPPs and POPs), whereas new emerging pollutants such as pharmaceuticals or per and polyfluoroalkyl substances (PFAS) have not been monitored or assessed as exhaustively or routinely (Geissen et al. 2015, Folorunsho et al. 2023). If more MPs are monitored and detected with low detection limits, the status of water bodies may not be considered as good. On the other hand, if MPs are not monitored at all or if the concentrations remain below analytical detection limit, the status can be considered as good, since no data is available (Loga and Przeździecki 2021). Regulations and monitoring activities are continually updated as our understanding of the behavior, ecological effects, and environmental fate of MPs deepens. However, our still very limited knowledge of these MPs makes modeling their environmental fate challenging.

### Model Uncertainty in Permitting Process

When models are used as decision-making tools it is crucial to appropriately assess the inherent uncertainty in the model. However, the natural unpredictability and high uncertainty that comes with model assessments stands in contrast to the need for predictability and certainty (Karkkainen 2002), which are essential elements in any well-founded legal structure. This presents a challenge in aligning scientific findings with legal requirements. The precautionary principle plays a crucial role in this context. It provides a guideline for decision-making in situations where scientific knowledge is uncertain. The precautionary principle, (De Smedt and Vos 2022) which manifests itself as the factor of safety in engineering, plays a central role in certain legal systems in the EU, where it has been made a statutory requirement in some fields of law. The precautionary principle, fundamental to fields like healthcare, trade, and environmental protection, guides policymaking even when full data is unavailable. It asserts that protective measures should not be delayed in potential risk situations, despite the absence of complete scientific certainty about the impact of proposed actions. When applying the precautionary principle, significant or misunderstood uncertainty can lead to a situation in which an uninformed decision is made purely due to limited understanding.

In Finland, the precautionary principle, combined with uncertainty in modeling, has been a significant factor in the rejection and postponement of large-scale industrial investments particularly in the context of ecological impacts. A notable example of this is the Finnpulp case in which the Supreme Administrative Court of Finland (SACF) denied an environmental permit for a planned pulp mill near a lake, adhering to the precautionary principle and model uncertainty. The modeling failed to demonstrate that the discharge from the planned pulp mill, including sulfates, chloride, and sodium, would not impair the good ecological status of the lake (SACF 2019). In its ruling, the SACF highlighted the lack of sufficient certainty that the proposed action wouldn’t compromise the lake’s good ecological status. In other words, the level of uncertainty was too high to grant a permit (Paloniitty and Kotamaki (2021), Thorén et al. 2021). Another environmental permit (SACF 2022) for a new mine was denied due to a poorly performed risk assessment of extreme events (e.g., excessive rainfall and drought) that could cause the prevailing conditions in a receiving water body to deteriorate and thus endanger the trout stock.

## Micropollutants in the Environment

The quality of a dataset needs to be assessed before it can be used for modeling. Several physical, chemical, and environmental factors affect the behavior of MPs and their measured concentration levels are reflective only at that instant sampling moment. The processes that affect the studied substances must also be considered before the dataset can be processed using ML techniques.

MPs are chemicals that are typically present in the surface waters at low concentrations and may be toxic to living organisms (Emadian et al. 2021). According to the REACH Regulation (EC 2006b), an MP is defined as persistent if its half-life in fresh water is over 40 days, and over 60 days in marine water (ECHA 2017). The environmental fate of an MP depends on its chemical and physical properties (Samiullah 1990). Furthermore, functional aspects of the molecule, electrical charge, biological activity, and concentration levels can affect the environmental fate of chemicals, as toxicity impedes biodegradation (Oliveira, Santelli (2010)).

MPs end up in watercourses via various but mainly anthropogenic sources. Wastewater treatment plant (WWTP) effluents, industrial effluents, scattered loading, and stormy waters can release MPs into rivers, streams, and lakes, where they end up in organisms and sediments or travel far away from their source with flowing water or air and eventually reach seas and oceans (Li et al. 2021). MPs can accumulate in fish and other aquatic organisms via bioaccumulation, biomagnification, or ingestion (Mostofa et al. 2013). In bioaccumulation chemicals are concentrated to organisms from aquatic phase, and in biomagnification the enrichment of chemicals occurs through trophic levels being higher in organisms on top of the food chain.

Modeling of anthropogenic MP loads in rivers requires information about the consumption, metabolism, removal rate at WWTP and degradation rate in the aquatic environment. However, the removal percent of e.g., pharmaceuticals ketoprofen and sulfamethoxazole can vary from 5–99% at the same WWTP and degradation rate of diclofenac in river conditions can even vary four orders of magnitude (Verlicchi et al. 2014, Gimeno et al. 2017). Many pharmaceuticals and other MPs also form conjugates, which cannot be detected with analytical techniques used for measuring original MPs. (Gewurtz et al. 2022) As pharmaceutical is excreted from human as conjugate and if it deconjugates at WWTP or receiving waters, the concentration in effluent is higher than in influent. The deconjugation can vary between WWTPs and depends on environmental conditions including e.g., enzymes present at treatment process.

### Micropollutants in Computational Modeling

**Fig. 2:**
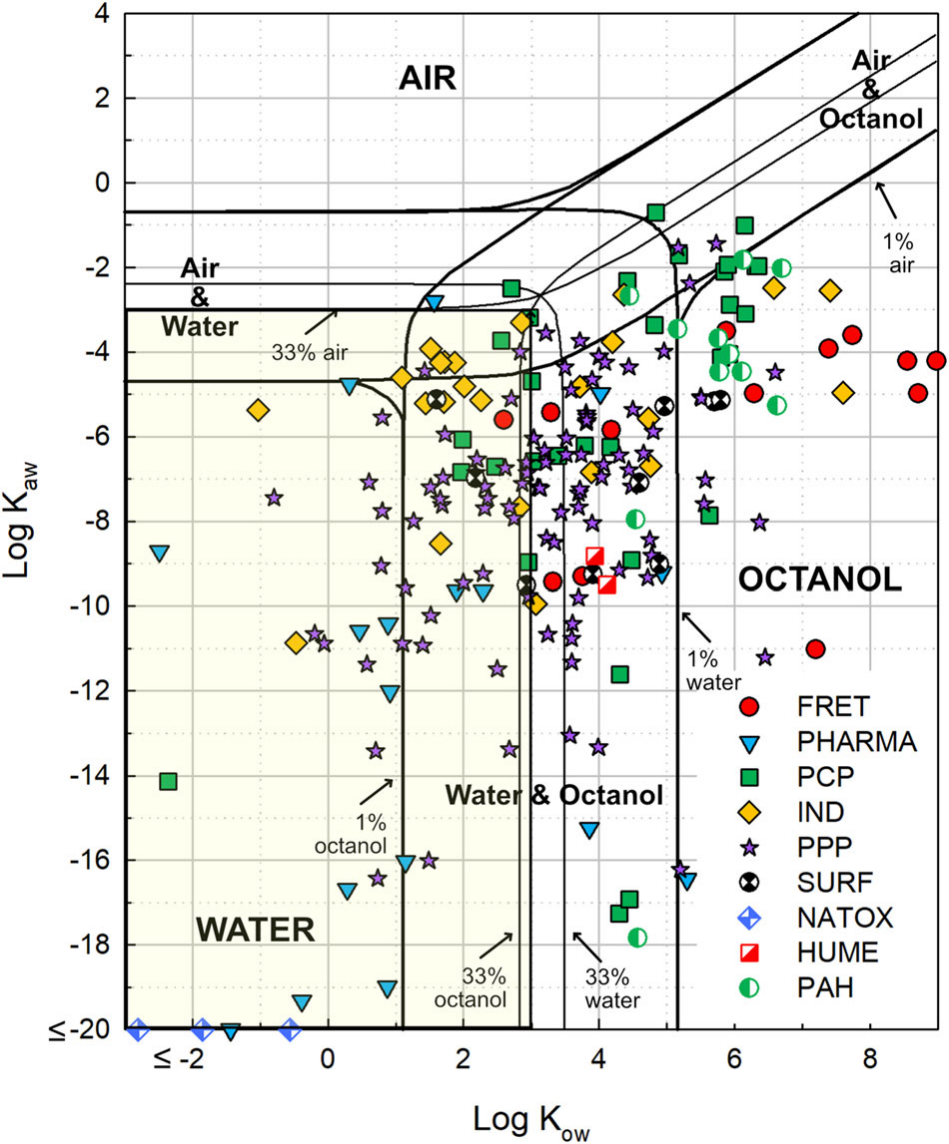
Water–air–octanol partitioning profile of measured MPs. MPs in the yellow box may have a high frequency of detection in the water phase of a river. (FRET Fire retardants, PHARMA pharmaceuticals, PCP personal care products, IND industrial chemicals PPP plant protection products, SURF surfactants, NATOX natural toxins; HUME human metabolites, PAH Polyaromatic hydrocarbons)

The development and use of computational modeling are easier when applied to chemicals with similar physico-chemical characteristics than chemicals that are markedly different. However, chemical groupings are usually based on performance or use and not physico-chemical characteristics. This can be based on the chemical analysis, which in study or monitoring projects is generally done for certain MP groups e.g., PFAS-compounds or pharmaceuticals. The chemicals of the same group can have dissimilar characteristics and due to that be present in different environmental matrixes e.g., compartments (Fig. 2). This makes the development of computational modeling techniques difficult, as almost every chemical within a subgroup (e.g., pharmaceuticals) must be considered as an individual. More difficulties are encountered, as different properties of MPs determine what processes are critical in modeling and which MPs need complementary information.

**Fig. 3:**
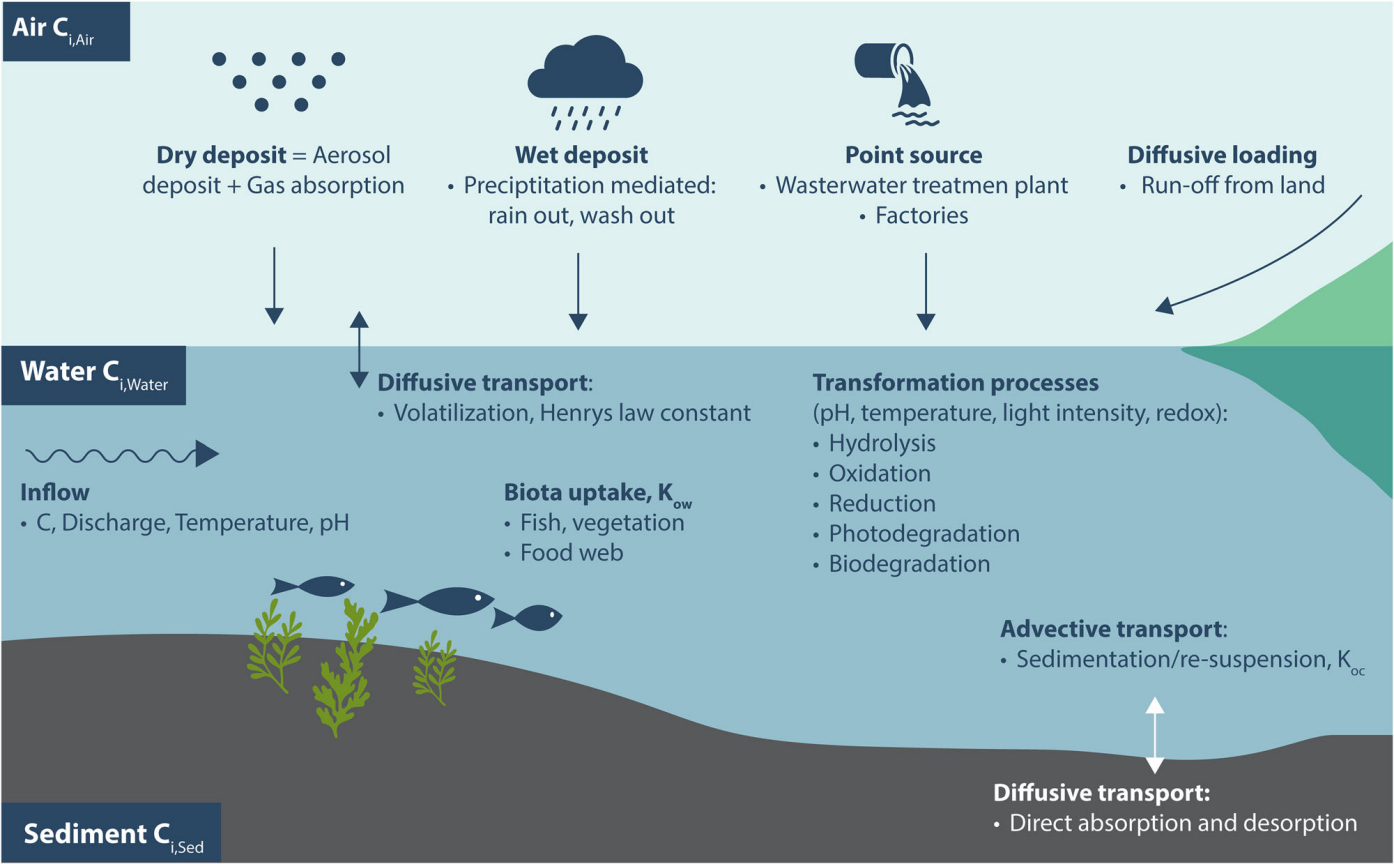
Processes involving MPs in the environment

The movement of chemicals between different environmental compartments, such as air, water, sediment, and biota, the interfaces need to be understood to predict their aquatic fate (Allan 1994). The behavior of MPs depends on the interaction between MP molecule and the environmental compartment. (Van de Meent et al. 2011) Chemicals can have very different transportation rates because of their physico-chemical characteristics (Fig. 3). Environmental conditions, such as temperature, wind, and precipitation, also affect the transportation process. In addition to concentration, bioaccumulation is also affected by lipophilicity, sedimentation rate, trophic level, and food webs. Furthermore, water residence time and the amount of particulate matter affect the environmental fate of MPs (Allan 1994). The transportation of chemicals does not eliminate them; instead, they are relocated to new locations that can even be in a different compartment. The transportation and transformation of chemical mass flows regulate the concentration levels of chemicals in the environment and in different compartments.

The transportation between different environmental compartments occurs via diffusive and non-diffusive processes (Parnis, Mackay (2021)). Diffusive transfer processes of chemicals include volatilization from water to air, uptake from water into a fish via respiration and adsorption, and absorption from water into sediments. If the phases are in equilibrium, the net transport rate of transfer is zero. Non-diffusive processes include rainfall from air to water, sedimentation of particles including chemicals, resuspension from sediment into water, and absorption into fish and biota via ingestion (Parnis, Mackay (2021)).

Chemicals can end up in watercourses via precipitation and rainfall, or volatilization into the atmosphere. E.g., Oliver (1984) observed that 80% of chlorinated benzenes from industrial and municipal sources are removed from lake environments via volatilization. Hydrophobic chemicals tend to be in the microlayer on the surface of a water body and in the interface between water and air; hence, their concentration is higher at the surface than within a water column. Due to the continuous transportation of volatile chemicals, they are found in aquatic environments (Allan 1994).

### Sampling

Sampling can be a significant source of error when studying MP concentrations in the environment. The sample must be representative, and contamination of the sample during and after sampling must be carefully avoided. Using clean sampling bottles and vessels, and avoiding contamination caused by perfumes, fuels, and surface finishing chemicals in clothing are important.

In general, MP concentrations in aquatic environment are measured in grab water samples taken from the study site at a certain moment which represents the MP concentration at that instant time of sampling. If MP concentration fluctuates, a grab water sample might be taken during high or low concentration (Vrana et al. 2010). If the concentration of MP in grab water sample remains below the detection limit, it does not necessarily mean that the MP is not present at the study site (Ahkola et al. 2022). Hence, the detected MP concentrations may not describe the actual prevailing conditions. Grab water samples can be taken more frequently e.g., once a day, but generally the bottleneck is the time-consuming and expensive chemical analysis of MPs. The monitoring of organic MPs in national monitoring programs is scarce, due to e.g., expensive analysis costs. Operators having environmental permits are obligated to monitor certain substances in the receiving waters generally 3–12 times a year, depending on the monitoring plan. However, with fluctuating concentrations monitoring with few samples can give misleading picture of the prevailing concentrations.

Grab water samples contain both the particle-bound chemical and dissolved fractions of the chemical. Environmental quality standards (EQSs) are used to estimate the status of a water course, and they are normally based on the total chemical concentrations. (EC 2000, 2008) The exceptions are nickel, cadmium, and lead, for which dissolved concentrations are used, as well as nickel and lead in inland waters, for which bioavailable concentrations are applied. Integrative sampling approaches such as composite sampling or passive sampling provide more accurate knowledge about average concentrations and the presence of MPs. Passive samplers collect MPs in watercourses for specific periods ranging from days to weeks. Passive samplers give an average concentration of MP during the deployment, discarding high and low peak concentrations. Furthermore, by integrating low MP concentrations to measurable level, passive sampling can recognize MPs which remain below the detection limit in grab water samples. However, passive samplers measure only the dissolved chemical fraction and hence, comparison with grab sampling results is not straightforward.

### Chemical Analysis

In general, MPs are extracted from sample matrix (water, sediment, and biota). The extract is then cleaned, concentrated, and analyzed using a suitable analyzing technique—which, in the case of organic MPs, is generally gas/liquid chromatography equipped with a mass detector. Sample pre-treatment can also introduce errors. MPs can form weak or strong bonds with sample matrix and extraction of strongly bonded MPs require more efficient extraction techniques than weakly bonded ones to reach high recovery (ECETOC (2010)). However, strong extraction and cleaning techniques can degrade MPs and decrease the recovery.

Commercial laboratories have accredited their analysis methods, but with a complex matrix, the detection limits can be higher. If a method of analysis is accredited, the quality of the analysis, including the sample storage and the condition of the instruments and reagents, is continuously supervised and accredited by external operator. The error in the concentration of organic chemical reported by an accredited laboratory to the customer is approximately 20–40%. The high uncertainty in chemical analysis can complicate the observation of differences between transformation, degradation, and other competitive processes. To lower the uncertainty, the chemical analysis of different monitoring campaigns should be conducted in the same laboratory. Then the results can be more reliably compared if the uncertainty can be considered similar for all the analyzed samples.

### Automatic Water Quality Sensors

Automatic, high-frequency sensors measure hydrological parameters (like discharge and water level) and traditional water quality characteristics, such as pH, electrical conductivity, turbidity, and TOC at a high temporal resolution, e.g., once or twice per hour (Kämäri et al. 2020). This is far more frequent than e.g., in general environmental monitoring water chemistry characteristics are measured few times a year. Still, only a few reliable web-enabled instruments for measuring MP concentrations exist (Post et al. 2022). Indirect detection of MPs with commercial online sensors can be performed by e.g., monitoring tryptophan concentrations online with an automatic sensor. (Sorensen et al. 2020, Briciu-Burghina et al. 2023) Tryptophan is an amino acid used in recognizing wastewater releases to surface or drinking water.

It is critical that the sensor data to be used in process models or statistical methods is of high quality and without errors. Suitable reliable sensors, correct installation, careful calibration, and sufficient maintenance ensure that the produced sensor data meets the target quality standards. However, erroneous measurements remain in the data, and these can be automatically filtered out or flagged for further inspections. In some situations, these errors can be identified via anomaly detection (Chapter “Extreme events and anomaly detection”).

### Uncertainties Related to Micropollutants in the Environment

Measuring the environmental concentrations of MPs contains several steps which can cause uncertainties to the results. Sampling, timing of sampling, sample pretreatment and analysis can all be vulnerable and depend on prevailing conditions as well as characteristics of MP. The sample pre-treatment and analysis are not similar for all MPs, so the uncertainties vary as well. Traditional water quality characteristics can be measured in laboratory much more accurately or even on sampling site with online sensors.

Uncertainty related to processes of MPs are largely dependent of the uncertainty of the concentration measurements and physico-chemical characteristics of MPs. The degradation and transformation can also depend on the prevailing conditions in e.g., WWTP. Also, the rates of transportation processes between environmental compartments depend on the concentration level of the chemical in the various compartments and the probability of the process occurring (Van de Meent et al. 2011). Therefore, uncertainty of any derived quantity is greater than uncertainty of the original measurement values (Taylor and Thompson 1982).

## Different Modeling Approaches

Modeling has been used in water management, impact assessments, and permitting for decades. There are several different modeling approaches to choose from, and the choice depends on the purpose of the modeling (large-scale management or more local permitting). Spatially or temporally coarse models perform best when applied to broad assessments while finer resolution for impact assessment and permitting. Temporally or spatially finer models require extensive amounts of measured input data and can be too computationally expensive for broad assessments.

The ever-growing trend of using computational modeling in environmental decision-making creates a high demand for accuracy and advanced uncertainty analysis in modeling. However, models cannot consider all relevant processes that leads to structural uncertainty and makes a comprehensive estimation of the uncertainty challenging.

Upon thorough examination of traditional physics-based modeling schemas, there is increasing pressure that they would being replaced by machine learning approaches such as physics-informed neural networks (PINNs) or long short-term memory (LSTM) neural networks. The reason is quite simple they perform better like LSTM and/or solve underlying equations faster (PINNS) thus enabling more comprehensive studies. However, the challenges limiting traditional modeling schemas remain unsolved.

### Process-Based Physical Modeling

Process-based physical models are based on first principles such as conservation of mass or energy. Several modeling approaches are available depending on the targeted accuracies and the targeted spatial and temporal scales. These models can capture fundamental phenomena with relatively sparse data. However, achieving accurate results necessitates knowledge of the physico-chemical characteristics of the studied substances. Additionally, constructing such models requires expert knowledge of the subject and sufficient time for modeling. Spatial and temporal scales that can be modeled via process-based physical model range from local to global and from tenth of a second to decades or even centuries if needed. However, the model suitable to global (decades) examination can rarely be utilized in local cases (tenth of a second). Only spatially and temporally coarse models can consider all compartments without becoming too complex and computationally heavy. Therefore, selection of approaches exists for different scales.

#### Multimedia fate modeling

Predictions of the environmental fate of MPs and their concentrations in different matrixes (e.g., water, land, and air) can be estimated based on MP characteristics, such as solubility, vapor pressure, and polarity (Samiullah 1990, Mostofa et al. 2013).

In multimedia fate modeling, the concentration of MPs in different compartments is presumed to be evenly distributed. The compartments typically considered are water, air, sediment, soil, suspended solids, and aquatic biota. The multimedia fate modeling approach delivers good performance when used to assess the overall environmental fate of a new MP at the level of policy formulation or when considering transnational cooperation to restrict the use of that chemical. These kinds of conceptually and computationally easy method can be linked or used as a life cycle assessment (LCA) methodology (ISO 2006, Mayo et al. (2014)). Recent customized spatially resolved multimedia fate models with smaller and more open spatial scales have become more conventional. When multimedia fate modeling is used on a smaller scale, it should be linked to intermedia (water, air, or ground water) transport models. However, combining multimedia fate modeling with either hydrological modeling (Lindim et al. 2016) or hydrodynamical modeling is a very promising approach.

##### Fugacity modeling

One application of multimedia fate modeling is fugacity-based models, also known as Mackay models (Mackay 1991), which describe the distribution of chemicals in various environmental compartments (Van de Meent et al. 2011). Using fugacity instead of chemical concentration enables the description of the chemical’s behavior using mass balance calculations and simulation of the transportation of released chemicals between compartments (Wang et al. 2020). In the Mackay models, the solid-water partitioning coefficient of the MP is estimated from its octanol-water partition coefficient (K_OW_). In general, this favors organic compounds for which K_OW_ that has been accurately estimated. Applying this approach to modeling polymers, inorganic chemicals, ionizable chemicals, or metals requires adaptation, which is achieved by considering their physico-chemical characteristics (Van de Meent et al. 2011). Fugacity modeling has been applied e.g., to assess the fate of estrogens in reservoirs receiving recycled wastewater (Cao et al. 2010).

#### Intermedia transport modeling

The environmental fate of chemicals is typically modeled only within one of the major compartments (i.e., water, soil, or air), which can be divided into smaller parts. Describing the intermedia transport of chemicals inside the compartment usually involves modeling the dynamics of the medium (i.e., water, or air). For water, this can be done using either hydrological or hydrodynamical models, depending on the desired modeling accuracy. The movement of chemicals between compartments in a single compartment model is considered based on the boundary conditions. Chemical processes can be modeled by formalizing the underlying physico-chemical phenomena (Bergström 1991, Chau 2010) discussed in Chapter “Chemical analysis” Due to the complexity of the processes that affect the transport of MPs within one compartment, rigorous deterministic modeling is, in most cases, impossible. Even if the MPs are dissolved in water, the deterministic model requires information on the concentrations in open boundaries and the transport of MPs between compartments. The problem of missing input data for hydrodynamical models can be mitigated e.g., by chaining them to hydrological models, or by using only coarser scale models.

Although the processes which affect transport are generally well recognized, most of them are usually neglected or not described as accurately as possible. This of course causes structural uncertainty. The processes are described in a model as either sources and/or sinks (water uptake) or boundary conditions.

##### Hydrological modeling of micropollutants

Hydrological modeling aims to simulate the movement and quality of water by simplifying the involved processes to the point where calculation is computationally feasible in a larger spatial domain. A hydrological model can be some specific part of the hydrological cycle (e.g., a lake or a river), or an attempt to model an entire water cycle system, starting from runoff after rainfall and ending at the point where river water flows into the ocean. Many hydrological systems use weather as a separate input, but there are also integrated modeling systems in which water transport in the atmosphere is included in a complete hydrological cycle model. A hydrological system may contain many water storages, such as basins (lakes), groundwater, snow, and surface retention, in which the behavior of water needs to be modeled to describe the system accurately. This can rapidly become computationally extremely heavy. Some, or all, parts of the system are parameterized to describe complicated processes using a simple relationship between some variables. In hydrodynamical or hydrological models, water sources or sinks must be added directly. Every industrial operator or WWTP that uses river or lake water, or discharges effluents must be included in the model. To build a hydrological model that predicts contaminant transport in rivers accurately and computationally inexpensively, it is necessary to make the model as simple as possible, but still accurate enough to predict a correct response of the variable.

Using hydrology, heat, and a solute transport model, Young and Fry (2020) simulated the transportation of pesticides from agriculture. The input data for the model included daily precipitation, temperature, wind speed, and pan evaporation. The water balance study included runoff, evapotranspiration, irrigation, and precipitation. Runoff, erosion, degradation, volatilization, leaching, dispersion, sorption, removal by plant uptake, and foliar wash off were used to calculate the vapor-phase and dissolved and adsorbed pesticide concentrations in soil.

##### Hydrodynamical modeling of micropollutants

A common way to numerically simulate a water body is to divide the system into small enough pieces for which the governing equations are solved in short enough time scales (i.e., discretization) that satisfy the stability criteria and the required accuracy. This approach is called hydrodynamical modeling, and the motion of the fluid is described as Navier–Stokes equations. Depending on the actual target (e.g., lake or river), different approximations are made for the Navier–Stokes equations before solving them; for example, Saint-Venant equations were used for the so-called shallow water approximations. For lakes, on the other hand, the Boussinesq approximation has been developed, i.e., density differences are neglected unless they appear in terms multiplied by the acceleration of gravity.

There are many solution techniques for Navier–Stokes equations, the most common being finite elements, finite difference, and finite volume methods. Running a conceptual (hydrological) or process-based model can be computationally expensive, and the results are still limited by the choice of parameterization and the accuracy of input data.

Fonseca et al. (2020) used the hydrodynamic particle tracking model (i.e., the Lagrangian approach) to study surface dispersion and the distribution of pharmaceuticals in estuarine environments. The model applies hydrostatic equilibrium and Boussinesq and Reynolds approximations and solves three-dimensional incompressible primitive equations. Simulations were performed for three flow scenarios (high, medium, and no flow at all) to take tidal effects into account. The main sources of pharmaceuticals are WWTPs and production facilities. The pharmaceuticals considered had different half-lives and degradation rates. Environmental fate studies of pharmaceuticals focus primarily on the sorption kinetics of only a few persistent pharmaceuticals, assuming first-order degradation kinetics. Abiotic conditions, such as salinity and pH, affect the electrostatic properties and sorption of pharmaceuticals to organic matter, leading to sedimentation (Fabbri and Franzellitti 2016). However, more concentration data are needed to apply more complex modeling (Fonseca et al. 2020). This same model has been used to estimate metal transport in salt marsh areas, with Duarte et al. (2017) observing that legacy metals are highly concentrated in halophyte vegetation, and mobilized detritus of over 200 kg of metals per year is released into estuaries. It appeared that neap tides keep the detritus in the inner estuary, but spring tides export them to the ocean.

### Data-Driven Methods

#### Machine learning methods in micropollutant modeling: Current status and research directions

At times, the requirements for building process-based physical models prove too stringent, hindering an exact understanding of the physical parameters of interest and introducing inherent uncertainties. In general, a lack of knowledge on physical phenomena or lack of time that is required to build a physics-based model for a specific environment can be partly compensated with more data and the right type of models, i.e., Machine Learning (ML) models that exploit large amounts of data to learn how to mimic the data generation process. One limitation to use ML in assessing environmental fate of MPs, is the lack of measured concentration data. However, indirect online sensor techniques can solve this issue (Chapter “Automatic water quality sensors”).

Machine Learning (ML) is an umbrella term that encompasses various approaches that address distinct aspects of learning from data. In the context of water quality or micropollutant modeling, supervised ML methods are the most relevant ones. Supervised ML methods are data-driven methods, where a model is trained with a labeled dataset, where each datapoint is associated with a corresponding output. In the context of water quality modeling, a typical example of a datapoint is a collection of measurements at a specific time such as temperature, runoff and pH, and a typical example of output is some water quality or micropollutant variable at the same time that is hard to measure such as E. coli level or residue concentration of pharmaceutical. The goal of supervised ML is to learn a mapping (i.e., model) from the datapoints to the outputs so that the model can be used to make predictions about the output given a datapoint. (Bishop 2006, Tahmasebi et al. (2020))

ML approaches started to popularize in hydrology after 2016 (Tao et al. (2016), Song et al. (2016)) with the rise of Deep Learning (DL), where deep Neural Network (NN) models are in central part. Some call this recent era as proof-of-capability era (Shen and Lawson 2021). NNs are layers of interconnected nodes, and deepness refers to a high number of layers. Each node in each layer is a simple tunable mathematical function that transforms its input (outputs of the connected nodes in the previous layer) into an output. The complexity and nonlinearity of deep NNs come from them chaining multiple simple mathematical operators. (Goodfellow et al. (2016)) DL models have been able to achieve great results on many hydrological prediction tasks, especially long short-term memory (LSTM) -models, that process sequential data by maintaining and updating an evolving hidden latent state. LSTM models for rainfall–runoff prediction typically achieves Nash-Sutcliffe efficiency (NSE) coefficient scores that are on par or better than those of their physics-based counterparts (Fang et. al. 2017, Kratzert et al. 2018, Gauch et al. 2019). LSTM models have also many applications in water quality modeling. Wang et al. (2017) and Liu et al. (2019) have used a LSTM model to predict water quality parameters such as dissolved oxygen. Baek et al. (2020) combined a convolutional NN (CNN), which is a model architecture for images and sequential data, with a LSTM model to be able to use rainfall radar images to predict water level and water quality. Convolutional NN has also been used to pre-process non-image data in a matrix format before feeding it as a latent input vector to a LSTM that predicts water quality (Barzegar et al. (2020), Yang et al. (2021)). LSTM models have also been recently used for predicting MP concentrations. Yun et al. (2021) used LSTM and CNN to predict six different MPs, such as acetamiprid, in a watershed. More recently, Tran et al. (2023) used LSTM and other ML models to predict microplastics in peatlands.

Graph neural networks (GNNs) have emerged as efficient and accurate data-driven models for rainfall–runoff prediction at multiple sites. By leveraging the inherent graph structure of a river network, these models exploit physical constraints and knowledge in the training phase. Recent studies have demonstrated the potential of GNNs for vector-based river modeling (Sun et al. 2021, Chen et al. 2021). GNNs have also been applied in water quality modeling. Li et. al. (2024) used GNN to predict pollution sources in a water distribution system and Santos et al. (2023) use GNNs to predict chloride concentration in urban streams. To our knowledge, GNNs have not yet been applied on MP prediction, but they could be used in similar applications as LSTMs if the data is structured in a graph like format, like in rivers.

Third DL approach that has provided promising results in hydrological applications is physics-informed neural network (PINN) (Raissi et al. (2019)). PINNs encode partial differential equations or other model equations into the neural network itself thus guiding the solution with known physico-chemical characteristics. PINNs have been a target of active research in recent years and there exists extensions that address various issues of the original PINNs such as computational efficiency (Kharazmi et al. (2019)), or nonlinear conservation laws (Jagtap et al. (2020)). PINNs have also been increasingly applied in hydrology. Especially different subsurface flow problems (He et al. 2020, Tartakovsky et al. (2020), Zhang et. al. 2022) have gained popularity in hydrological applications in recent years. Water quality modeling related applications are not yet as numerous, but there exists a growing number of research, for instance from pollution transport modeling (Bertels and Willems 2023), source modeling (Tang et al. (2023)) and water quality parameter prediction (Jahanbakht et al. (2022)). Like GNNs, PINNs do not have applications for micropollutants. Potential use cases are many, as the physico-chemical processes of many microplastics are at least partially known. Development of PINNs benefit from multidisciplinary cooperation of physicists, chemists and machine learning specialists. Typically, ML models behave like black boxes in the sense that they do not provide an explanation for why they generate the predictions they do. Unknown physico-chemical processes that are present in the data generation process will stay unknown with these models. In other words, these models generally do not reveal anything previously unknown about the internal physico-chemical processes that affect how the data has been generated. There do exist ML approaches that can be used for differential equation discovery (Rackauckas et al. (2021)), but these approaches yet work for relatively simple systems with no noise and are not able to reliably find the true mechanistic equations. The authors have not found proof of these approaches being applied on water quality modeling and it is relatively safe to say that ML approaches applied on water quality modeling are black boxes.

ML models are generally weak at extrapolating since they are bound to the data that has been used to train the model. That is, if a ML model, such as a random forest or a simple deep neural network, has been trained on data on a specific river basin, applying that model to a different basin will probably yield poor results. Conversely, physical hydrological models typically perform better when applied to changing and unmeasured conditions, considering that assumptions about physico-chemical characteristics remain valid. For instance, if the current measured water quality is worse than the historical records, a ML model is likely to perform worse than a physical model. There is evidence that this claim might also hold true for the modern and more sophisticated data-driven models (de Moura et al. 2022). However, large-sample hydrology (LSH), which is aimed at understanding hydrological processes at multiple spatiotemporal scales and under changing conditions (Addor et al. 2020) is somewhat challenging this regime. The previously mentioned LSTM models (Kratzert et al. 2019) and GNN models (Sun et al. 2021) are examples of ML approaches that leverage vast amounts of hydrological data. Although LSH has not yet been applied in water quality or micropollutant modeling, they are promising directions of research. To provide applicable approaches, different water quality datasets must be combined such that they all have similar structures in terms of metadata, geographical description, and the actual time series data on water quality measurements. Recently, many research projects have been aimed at producing such datasets (Klingler et al. 2021, Kratzert et al. 2023), and more recently, some steps have been taken toward producing such data for chemical variables (Rotteveel et al. 2022, Ebeling et al. 2022, NORMAN 2023). Although LSH is able to make predictions for multiple spatiotemporal scales, it does not provide superior performance over other ML models for unseen data as is typical for surprising events and weather extremes, that are likely to become more popular due to the climate change (Stott 2016). Also, MPs are often not as commonly measured as some water quality parameters, making it harder to gather LSH datasets that would benefit micropollutant modeling.

#### Extreme events and anomaly detection

Water quality data —and MP concentration data in particular— are generally sparse and usually available only at a very limited sampling rate; On the contrary, hydrological parameters (e.g., water flow) can usually be measured more frequently and for longer periods of time.

Anomalies in hydrological parameters, like sudden changes in water temperature and flow, can lead to false positive (or false negative) detections in water quality. (Muharemi et al. 2019) That’s why it is important to monitor both types of data to compensate for these events.

**Fig. 4:**
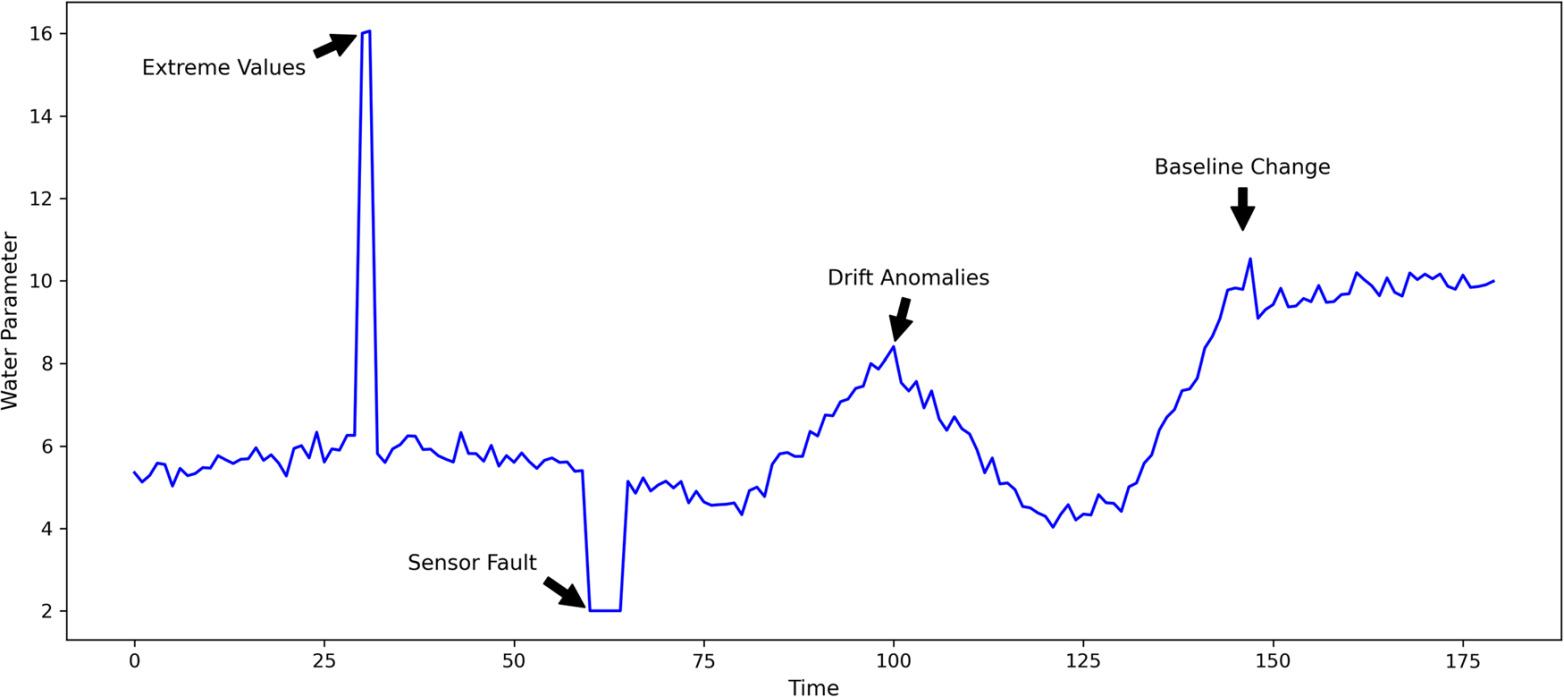
Difference of anomaly and extreme value

Given a time series of measurements, we can distinguish between two main types of events: extreme values and drift anomalies (Fig. 4). Extreme values, characterized by brevity and significant deviation from normal ranges, are critical indicators of potential catastrophic events or sudden and brief pollution episodes. These may include drastic increases in contaminant levels following industrial accidents or unprecedented changes in flow rates due to climatic extremes. Extreme values might also be due to sensor faults, in which case they should be masked or properly replaced using statistical imputations techniques.

Drift anomalies are gradual shifts in the data distribution, that can stabilize over time (baseline change) or drift back to the previous distribution. They may not reach extreme thresholds, but still signal pollution events or anomalies which are sustained for a long period of time. An example are sustained Increases in aquatic nitrogen and phosphorus due to urban and industrial activities, leading to eutrophication and threatening water quality and biotic integrity from headwater streams to coastal areas world-wide (Wurtsbaugh et al. 2019).

Xu and Liang (2021) highlight the increasing importance of machine learning techniques for modeling time series of hydrological and water pollution measurements. They apply machine learning models to detect anomalies in water pollution measurements, citing the imbalance between negative (normal) and positive (anomalous) examples as a main challenge for this type of approaches.

In hydrology and climatology, the concept of extreme events has been pertinent since the beginning of field study measurements. One reason for this is the contrast between lack of water (i.e., due to droughts and dry seasons) and abundance of water (i.e., via floods and heavy rains), both of which are extreme events. Gumbel distribution (Gumbel 1935, 1941) has been used since the 1950s to analyze return periods and other temporal characteristic features of extreme events. Return periods and levels are widely used when planning land use and infrastructure development. Recent advances have enabled the utilization of extreme value theory in tasks on identifying anomalies (Vignotto and Engelke 2020, Rudd et al. (2018)).

Statistical models are often applied to the detection and/or prediction of unexpected behavior, such as sudden changes in the quality of water. At simplest, these anomalies can be detected by looking at their F-scores. Unfortunately, one of the main challenges of this technique is a high imbalance in the data, which reflects a sparsity of anomalous events relative to normal events (Muharemi et al. 2019, Nicholaus et al. 2021). Several models and techniques have been applied to address this problem. Yu et al. (2014) applied a sliding window technique and an autoregressive model to detect outliers in hydrological time series. Comparably, Alim et al. (2020) applied the autoregressive integrated moving average (ARIMA) model with a moving window. Using an autoregressive linear combination model, Zhang et al. (2017) devised a dual-time-moving window system that can identify anomalies in real time using historical data. Furthermore, Kulanuwat et al. (2021) applied median absolute deviation (MAD) to estimate water levels. MAD is a robust scale estimator that detects anomalies and is insensitive to large deviations in the data but estimates the deviation from the median. Although these simple statistical methods are not considered to be ML models, they are data-driven and popular in real applications.

Many advanced ML approaches have also been tried for anomaly detection throughout the years, and different support vector machine (SVM) approaches seem to have good performance in the applications related to hydrology and water quality modeling. SVM is a relatively simple ML model, which combines nonlinear transformation to a linear model. Muharemi et al. (2019) applied a plethora of data-driven models to detect anomalies in water quality parameters and found that SVMs and NNs yield the most optimal results. By testing the pretrained models on different datasets, it was found that the logistic regression approach and SVM formulation were the most robust approaches in situations where there is a shift and/or change of scale in the data distribution. Another notable study on the implementation of an SVM model was performed by Tan et al. (2020). They designed a dual-stage one-class SVM for water level monitoring, first detecting pointwise anomalies in time and then detecting patterns generated by collective anomalies in a second stage. Shi et al. (2023) compared four different approaches in detecting anomalies. SVM based One-Class SVM was detected to have the best detection performance.

Other approaches exist as well. Chachula et al. (2021) devised and implemented a data fusion system capable of exploiting multiple time series sensor measurements to efficiently localize pollution sources. Jiang et al. (2020) analyzed anomalies in the dynamics of river water quality parameters, such as water temperature, pH, dissolved oxygen (DO), conductance, turbidity, nitrate plus nitrite (NOx-N), and discharge. They used a combination of Fourier and wavelet spectral analyses of high temporal resolution measurements of the Potomac River in the USA. An example of another recent and novel approach is a study on anomaly detection for water levels performed by Nicholaus et al. (2021) using a deep autoencoder network: a network that learns to reconstruct the input from a latent and compressed vectorial representation. When the reconstruction error is above a certain threshold, the event is classified as anomalous. Yet another novel usage of ML for anomaly detection in hydrology has been developed by Dias et al. (2020), taking advantage of images from remote sensing and incongruence-based anomaly detection to detect water pollution in the surface waters of open water bodies and large rivers.

### Uncertainties in Modeling

#### Process based modeling

When using computational models to predict the environmental impact of e.g., industrial investments and environmental management, the accuracy of the modeling results with respect to measured data must be assessed (Beven and Alcock 2012). In such an assessment, the uncertainty of modeling and the uncertainty of measured data must be considered simultaneously. For dispersion studies of MPs this is problematic since chemical measurement from the study area are sparse and thus insufficient for the comparison (Chapters “Sampling” and “Automatic water quality sensors”). As stated in a chapter “Intermedia transport modeling”, intermedia transport of MPs relies on the fluid transport within a media, and in practice, an intermedia fluid transport model itself is often calibrated, validated, and verified to provide information on how much the modeled parameter (e.g., average speed magnitude, direction or discharge) deviates from the measured values. Different metrics and indicators for comparing modeling results and measured data are extensively considered in the scientific literature (Ferreira et al. 2020). However, the literature of uncertainty analysis regarding hydrodynamical modeling is sparse when compared to hydrological modeling. One of the reasons might be the fact hydrodynamical and hydraulic models are used in many engineering or scientific sectors and for that they may need different performance metrics or evaluation tools (Alexandrov et al. 2011).

One of the basic rules of error analysis is that uncertainty will never decrease when introducing a new variable (or data) and its errors. When considering simulations (e.g., solving time-dependent differential equations in situations with open boundaries), more and more uncertainty is generated in the system on each time step. Explicit analysis of error propagation within simulations is either extremely difficult or poorly performed, which leads to an unlimited increase in uncertainty during simulations. Despite the significant role of forcing data most of the uncertainty literature seems to concentrate on the uncertainty originated from the input parameters (Pinheiro et al. 2019) and influence of forcing data (Juntunen et al. 2019, Camacho et al. 2014) and its uncertainty is neglected.

When considering effect of the uncertainty of the forcing data, error propagation within system must be taken account (Kühne et al. 1997) Rigorous error propagation analysis for time-dependent simulations is complicated if even possible. To be able to use analytical error estimation, the equations describing the problem analytically must be solved (Thompson et al. 2008). In the case of hydrodynamical models, this is not possible; thus, different approaches are required. One approximative uncertainty estimation technique is the first-order second moment (FOSM) method, which mimics a method based on the error propagation law (Taylor and Thompson 1982) that can be used when the analytical solution of an equation is known. In the FOSM approach, the model itself acts as a known, true solution to the problem. This approach is justifiable only if structural error related to the model itself can assumed to be negligible. Another rather simple possibility is to use the Monte Carlo approach, in which distributions of random samples from input data are drawn and used as input data for the model. Techniques for doing such analysis is numerous (Camacho et al. 2015). This kind of approach is usually referred to as a global uncertainty analysis compared to the “classical” sensitivity analysis of an individual parameter (Mayo et al. (2014)).

While the uncertainty induced by parameter studies using the Monte Carlo methods is plausible for tens of thousands realization (combination of parameters), it is not plausible in a case of forcing data since computational burden would be too high. In practice analysis is limited to a few scenarios based on extreme situations and mean or median cases. Regardless of the modeling approach the most important source of the uncertainty seems to be the input data (Wang et al. 2020).

#### Machine learning methods

ML methods rarely provide uncertainty estimates for the model outputs without the need of modifying the model architecture. If uncertainty estimates are necessary, one should look for methods that support them from the beginning. As uncertainty estimates are often neglected in the research, there exists less approaches that are uncertainty aware and the model performance of these methods is usually worse in terms of prediction accuracy. There exist examples of uncertainty aware extensions to both LSTM models and PINNs in the context of environmental sciences (Klotz et al. (2022), Lütjens et al. (2021)). Estimating uncertainty of the model output is fast in general for all these aforementioned methods and in this aspect, ML approaches can be very compelling.

In addition to extensions to the previously introduced ML approaches, there exists other popular approaches for uncertainty estimation in ML in general. When considering uncertainty in relation to predictions (e.g., the confidence interval), ML-based methods offer computationally faster approaches than physical models.

Common approaches to estimate the prediction uncertainty of ML models include ensemble methods (Abdar et al. (2021)), Bayesian methods (Mena et al. (2021)), and conformal prediction (Angelopoulos and Bates 2023). Due to its simplicity, the Monte Carlo dropout technique has also been widely adopted (Gal and Ghahramani 2016). There are examples in the literature of data-driven uncertainty estimation applied to rainfall–runoff models (Shrestha and Solomatine 2008) and the prediction of river water quality index (Asadollah et al. (2021)).

## Comparison of Modeling Approaches in Sense of Uncertainty

It seems that both modeling strategies, traditional and ML, use quite similar methods to obtain uncertainties. While models can perform well according to one metric, they can perform poorly with some other metric. When using models to predict the environmental impact of e.g., industrial investments and environmental management, it is essential to assess the accuracy of the modeling results in relation to the measured data (Beven and Alcock 2012). In such an assessment, both the uncertainty of the modeling and the uncertainty of the measured data must be considered. Taking into account the precautionary principle, the most important uncertainty is associated with the predictions of the model i.e., prediction uncertainty. Both traditional modeling and ML based techniques can generate this type of uncertainty. However, ML methods typically produce them faster than traditional methods.

For traditional modeling as well as for ML methods the quality of the input data is crucial. For both methods uncertainty caused by the forcing data can be evaluated but for the ML approaches this happens much faster than for traditional process-based models. The difference in speed naturally depends on the speed of the underlying process-based model, and consequently, on the spatial and temporal resolution. The uncertainty of input data derived from chemical measurements and environmental behavior of MPs considers several steps These range from the timing of sampling, which is crucial when concentrations fluctuate, to the analysis and physico-chemical characteristics of the MPs. The uncertainties from MP analysis in commercial laboratory are considerably high being 20–40%. However, in environmental monitoring and legislation, these MP concentrations are generally treated as exact ones. If the uncertainty of modeling is compared with the uncertainty of MP concentration measurements, they may not differ that much. Hence the use of modeling in estimating changes in chemical status can be considered as reliable as laboratory analysis measurements, in case no measured input data exists.

## Tackling the Uncertainties of Input Data

To reduce the uncertainty of MP modeling the quality of the input data should be ensured (Fig. 1). Generally, the available input data is not collected solely for modeling purposes, but the data is rather a compromise of several goals related to monitoring (Biber 2013). Therefore, planning a sampling strategy which better serves modeling purposes will reduce uncertainty. Furthermore, by paying attention to certified sampling as well as accredited sample treatment and analysis, the reliability of the MP concentration data can be increased. The continuous development of online monitoring techniques will provide more MP concentration input data and improve the reliability of modeling. Also, analyzing the input data to remove possible outliers will diminish the uncertainty.

In rigorous traditional MP modeling that captures all known possible processes, more information is required (Fig. 3). This includes locations of sources/sinks (i.e., environmental compartments) as well as the values of parameters describing the processes associated with them. With MLM, the correlations between MP concentrations in different environmental compartments can be recognized (Fig. 3). This is based on the information included in the input data without the need for any additional measurements. In a way, the use of MLM reduces the number of required input data types, which simplifies data collection. However, this does not imply that the input data is not the largest source of uncertainty. It simply means that MLM can potentially make the process of reducing uncertainty easier.

MP concentrations can be indirectly determined by measuring the related water quality characteristics. These characteristics can be detected more easily with online techniques, for example, than the MP of interest. The uncertainty of conventional MP analysis is smaller than when converting water quality characteristic measurements to MP concentrations. However, as the latter practice provides more input data, the uncertainty is also reduced. Therefore, addressing the uncertainty of input data also involves certain compromises.

## Concluding Remarks

The implementation of the results of modeling, including uncertainty and the precautionary principle, should be researched more deeply to achieve a reliable estimation of the effect of an action on the chemical and ecological status of an environment without underestimating or overestimating the risk. Organic MPs are included in modeling cases, but the dearth of measured concentration data is generally a limiting factor. The growing use of computational models in environmental decision-making creates a high demand for accuracy and advanced uncertainty analysis in modeling. Process based models cannot consider all known and relevant processes, making the comprehensive estimation of uncertainty challenging. Problems in a comprehensive uncertainty analysis within ML approach are even greater. For both approaches, generic and common methods seem to be more practical than those emerging from ab initio. Regardless, the prevailing uncertainties need to be identified, acknowledged, and if possible, reduced. One approach to reduce uncertainty in modeling is to increase measurements, as the precision of input data directly impacts the model’s accuracy. As the volume of data continues to grow, Machine ML techniques may become increasingly vital for direct MP modeling. This is particularly true as emerging ML approaches, such as LSTMs and PINNs, continue to evolve and find new applications.

Currently, process-based modeling can provide reasonable estimations of the transport and fate of MPs in the environment at coarse spatial and temporal scales. This information is valuable for policymakers and governmental authorities when assessing the risks of water construction activities. For large-scale fate studies, the ability of ML to extract information from large databases, such as NORMAN (2023), could prove useful. At these scales, uncertainties are often not considered as seriously as they are in permitting processes. Overall, it is wise to agree with Rykiel (1996) and adopt pragmatic approach that “a model only needs to be good enough to accomplish the goals of the task to which it is applied.” (Rykiel 1996, Moriasi et al. 2007) Anyhow quite often situation is that each modeling strategy has its own advantages and disadvantages and modelers to be aware of them (Klügl and Kyvik Nordås 2023).

One could argue that the precautionary principle (see Chapter “Modeling and monitoring requirements”) serves as a guiding principle in modeling process aimed at permitting. Modelers make choices that, if not reflecting the absolute worst-case scenario, at least align with very conservative choices. These choices correspond to the sensitivity analysis of the forcing data (Paloniitty and Kotamaki (2021), Karl et al. 2011). This approach is one of the practical ways to address uncertainty in a modeling process. However, issues arise when these conservative selections lead to probable problems. In such cases, the permit is either rejected, or modeling is performed again.

Currently, ML methods are not used in the permitting processes (at least in Finland) due to the lack of data and their inability to perform spatial interpolation between measurement points. It is however matter of time when hybrid methods emerge where ML methods like PINN replace finite-difference or FEM/FVM methods to solve the underlying process-based equations (Haupt et al. 2022) This kind of approach would also solve the problem that ML currently has which is associated to the changing operational environment.

Coglianese and Lehr (2017) considered a situation where legal authorities use AI and ML. Their legal analysis suggests that they will likely face difficulties in implementing ML within existing administrative constraints. They identified three reasons why the reliance on ML may cause concerns for governmental authorities: self-learning ability, black box nature, and its speed. The last factor implies that the process might shorten or even bypass human deliberation and decision-making. One of the main conclusions drawn by Coglianese and Lehr (2017) regarding transparency in decision-making is that “well-informed, responsible use of machine learning can be compatible with long-established principles of accountable and transparent administrative decision making, even if some algorithmic components may be exempt from public disclosure.” They were considering a situation where an agency itself would use ML to make decisions, but the rationale should be similar when authorities accept evidence from third parties.

One reasons why authorities remain hesitant to incorporate ML into permitting processes is due to its “black box” nature (see Chapter “Intermedia transport modeling”). Workshops organized in several national projects (in Finland) have revealed that the acceptance of new methods is impeded by the requirement for authorities to justify their decisions. For ML, they struggle to understand how the methods arrive at their results (unpublished data).

To our understanding, both the CJEU and the SACF have not yet encountered a case where an environmental permit for a new polluting activity is denied, with the decision primarily based on the chemical status objectives. This implies that such a scenario, where chemical status objectives directly influence permit decisions, is yet to be legally tested. Drawing from the arguments presented in the Weser and Finnpulp cases as precedents, it is likely that similar situations will arise in the future. The application of modeling results, which includes elements of uncertainty and the precautionary principle, warrants further research. This is to ensure a reliable estimation of the impact of an action on the chemical and ecological status of an environment, without either underestimating or overestimating the risk. To conclude, the Weser ruling, in conjunction with the precautionary principle, underscores the significance of modeling uncertainties, at least in the context of Finland (Paloniitty 2016, SACF 2019, Paloniitty and Kotamaki (2021)).
